# Diagnosis, Therapy, and Prognosis for Hepatocellular Carcinoma

**DOI:** 10.1155/2020/8157406

**Published:** 2020-02-03

**Authors:** Zhigang Ren, Xiaochao Ma, Zhenfeng Duan, Xinhua Chen

**Affiliations:** ^1^Department of Infectious Diseases, The First Affiliated Hospital of Zhengzhou University, Zhengzhou 450052, China; ^2^University of Pittsburgh, Pennsylvania, USA; ^3^David Geffen School of Medicine at University of Los Angeles, Los Angeles, CA, USA; ^4^Department of Hepatobiliary and Pancreatic Surgery, The First Affiliated Hospital, School of Medicine, Zhejiang University, Hangzhou 310003, China

Hepatocellular carcinoma (HCC) is one of the most common malignancies worldwide and the third leading cause of cancer-related death worldwide [1]. Currently, there are an estimated 29,200 new HCC cases in males and 11,510 cases in females in the United States in 2017, among which 29,000 cases died from HCC [2]. More seriously, estimated new HCC cases achieved 343,700 in males and 122,300 in females in China in 2015 [3], which is mainly attributed to the prevalence of hepatitis B virus (HBV) persistent infection and HBV-induced cirrhosis. Due to the absence of specific symptoms in early stages, the lack of early diagnostic markers, and the low percentage (10–20%) of radical resectable HCC on diagnosis, most HCC patients are often diagnosed in an advanced stage with poor prognosis (the overall ratio of mortality to incidence is 0.95) [4]. Although liver transplantation (LT) has been considered the most effective therapy for HCC [5], organ shortage significantly limits the application of LT. Even if sorafenib and regorafenib are applied in HCC, the overall outcome of HCC remains unsatisfactory with the median survival in early and advanced HCC of about 6-9 months and 1-2 months, respectively. Thus, it is very important to explore the novel diagnosis tools, therapy strategy, and prognosis markers for HCC.

In this special issue, we present a delicate collection of high-quality reviews and original articles that contains the latest thinking and exploration for the mechanisms of HCC development, the biomarkers of HCC diagnosis and prognosis, and the therapeutic strategy of HCC. Full acquaintance with the diagnosis, therapy, and prognosis for HCC can be acquired through fundamental investigations and clinical research. The purpose of this editorial is to provide a brief introduction for each published or accepted paper and highlight their major findings and discoveries. We believe that these achievements will help to find a better way to deal with HCC, so as to conquer it eventually.

As HCC is a highly vascularized tumor, new vessel formation in the tumor plays an important role on HCC progression, metastasis, and recurrence. S. Hu et al. investigated the function of ginsenoside Rg3 on angiogenesis and explored the possible mechanism in an orthotopic murine model. The experiment results showed that ginsenoside Rg3 initialized the tumor apoptotic progress, which then weakened the tumor volume and its capability to produce the vascularized network for tumor growth and further metastasis. This study indicates clinical potential of using ginsenoside Rg3 in the angiogenesis therapy against HCC.

Instead of Rg3, M. N. Hasan et al. studied a novel Strigolactone (SL) analogue, TIT3. To reveal the mechanisms of TIT3-induced anticancer activity in HepG2, they performed RNA sequencing and the differential expression of genes was analyzed by different tools. Through the analysis of the experiment results, researchers demonstrated that the inhibition of HepG2 cancer cell growth was attributed to the interplay of genes, wherein the treatment of TIT3 significantly altered their expression levels. These altered gene expressions affected cell proliferation, cell cycle, metastasis, and apoptosis.

Locoregional therapies are increasingly available and gradually benefit many patients of HCC. Z. Xu et al. reviewed the characteristics and advantages of transarterial chemoembolization (TACE), radiofrequency ablation (RFA), microwave ablation (MWA), and TACE combined with RFA or MWA in order to provide the physician a better background on decision. The authors concluded that TACE combined with either RFA or MWA was effective and promising in treating larger HCC lesions. Most of the interventional operations needed local anesthesia combined with intravenous sedation. Q. Jin et al. summarized and analyzed multiple anesthesia methods and their characters while being applied in interventional therapy for HCC. These results suggested that different percutaneous ablations should be performed by selecting the appropriate anesthesia method to obtain the best therapeutic effect. For instance, as the prevalence of TACE, increasing clinical cases about post-TACE pulmonary complication had been reported. L. Fang et al. retrospectively analyzed 14 HCC patients who were diagnosed with TACE-associated acute lung injury (ALI). Pulmonary Lipiodol embolism was one of the main causes of TACE-associated ALI. Hence, precise evaluation, early recognition, and management are critically important during TACE.

Recently, the long noncoding RNAs (lncRNAs) are involved in many human cancers, including liver cancer. H. Shi et al. summarized the differences in the expression of lncRNAs in HCC and reviewed the participation of lncRNAs in HCC cell proliferation, apoptosis, and migration. The lncRNA might be a candidate biomarker for the diagnosis, prognosis, and recurrence prediction, as well as the therapeutic target of liver cancer. Analogously, M. Elfar and A. Amleh explored miR-590-3p and its potential downstream target genes in HCC cell lines and discovered that SOX2 could be a direct downstream target gene of hsa-miR-590-3p in HCC, signifying its role in epithelial-mesenchymal transition. They also indicated that the hsa-miR-590-3p downstream targets had huge research potential.

Another interesting research was reported in detail by V. G. Bychkov et al., who explored the patterns of the intensity of the invasion and egg production of Opisthorchis felineus in the carcinogenesis of various organs and partial hepatectomy in the setting of superinvasive opisthorchiasis. When modeling tumors with various carcinogens in the setting of superinvasive opisthorchiasis, the intensity of invasion was reduced. On the contrary, a partial hepatectomy in the setting of opisthorchiasis did not affect the number of parasites in the ecological niche (liver). This finding implied the potential impact of liver cancer on the body.

In view of the above review and discussion, we believe that the present special issue explores the latest research on the diagnosis, therapy, and prognosis for HCC. Indeed, we need to further explore advanced and effective diagnostic tools, safe and minimally invasive treatments, and convenient and sensitive prognosis assessment for HCC.

*Zhigang Ren* *Xiaochao Ma* *Zhenfeng Duan* *Xinhua Chen*
